# Prevalence and Trends of Adult Obesity in the US, 1999–2012

**DOI:** 10.1155/2014/185132

**Published:** 2014-01-06

**Authors:** Ruopeng An

**Affiliations:** College of Applied Health Sciences, University of Illinois at Urbana-Champaign, George Huff Hall Room 2013, 1206 South 4th Street, Champaign, IL 61820, USA

## Abstract

*Aim*. To provide national estimates of obesity among US adults aged 20 years and older in 2011-2012 and track its trends from 1999 to 2012. *Methods*. Measured weight/height from National Health and Nutrition Examination Survey 1999–2012 waves was used to calculate body mass index (BMI) and prevalence measures. Piecewise logistic regressions were conducted to test the differential trends before and after 2010. *Results*. In 2011-2012, the age-adjusted prevalence of overweight and obesity combined (BMI ≥ 25) was 71.1% (95% CI: 68.0%–74.2%) among men and 65.5% (61.8%–69.3%) among women, and the prevalence of obesity (BMI ≥ 30) was 33.3% (30.5%–36.2%) among men and 35.8% (32.3%–39.4%) among women. From 1990–2000 to 2009-2010, the prevalence of overweight and obesity combined, obesity, grades 2 and 3 obesity combined (BMI ≥ 35), and grade 3 obesity (BMI ≥ 40) increased by 7.2%, 17.8%, 17.6%, and 33.0%, respectively. Compared to 2009-2010, most gender- and race/ethnicity-specific prevalence measures remained unchanged or slightly decreased in 2011-2012. No significant difference in trends among prevalence measures was found before and after 2010. *Conclusions*. Concurrent evidence on the leveling off of obesity in the US is thin. Given its high prevalence and profound socioeconomic consequences, close monitoring of the trend is warranted.

## 1. Introduction

Obesity is a leading risk factor for many adverse health outcomes including type 2 diabetes, hypertension, dyslipidemia, coronary heart disease, and certain types of cancer [1]. The prevalence of adult obesity in the US had doubled from 1976–1980 to 1999-2000 [2]. In 2009 US ranked the highest in adult obesity prevalence among all countries in the Organization for Economic Cooperation and Development [3]. The estimated annual medical cost of obesity in the US totaled 147 billion US dollars in 2008 [4].

More recent data indicated a slowing down of the trend in adult obesity and even some leveling off in childhood obesity. Small but significant declines in the prevalence of obesity among low-income preschoolers aged 2–5 years were reported in 19 of 43 US states examined in the Pediatric Nutrition Surveillance System from 2008 to 2011 [5]. No significant change in the prevalence of adult obesity was found between 2003–2008 and 2009-2010 in the National Health and Nutrition Examination Survey (NHANES) [6]. The prevalence of morbid (i.e., grades 2 and 3) obesity was still increasing from 2005 to 2010, but its growth rate had slowed down as indicated in the Behavioral Risk Factor Surveillance System (BRFSS) [7].

Given the large disease burden of obesity and its high prevalence, it is crucial to continuously monitor the prevalence of obesity in the US. This study provides national estimates of obesity among US adults aged 20 years and older in 2011-2012 and tracks its trends from 1999 to 2012.

## 2. Methods

Study sample came from NHANES 1999-2000, 2001-2002, 2003-2004, 2005-2006, 2007-2008, 2009-2010, and 2011-2012 waves. NHANES is a program of studies designed to assess the health and nutritional status of adults and children and represents a multistage probability sample of the US civilian, noninstitutionalized population [8]. Participants' body weight and stature height were measured by digital scale and stadiometer in the NHANES mobile examination center [9]. Body mass index (BMI) is defined by weight in kilograms divided by height in meters squared. Four prevalence measures were examined: overweight and obesity combined (BMI ≥ 25), obesity (BMI ≥ 30), grades 2 and 3 obesity combined (BMI ≥ 35), and grade 3 obesity (BMI ≥ 40). Age was adjusted by direct standardization to the year 2000 Census population using the age groups 20–39 years old, 40–59 years old, and 60 years and older. NHANES wave-specific sampling weight, sampling stratum, and primary sampling unit were taken into account in estimating prevalence in the population. Prevalence was estimated for both the overall population and subpopulations stratified by gender and race/ethnicity (i.e., non-Hispanic White, non-Hispanic African American, and Hispanic).

Piecewise logistic regressions were used to test the differential trends in adult obesity before and after 2010. The model has the following setup:

(1) $$\begin{array}{c} \text{logit}\left(Y_{i}\right)=\beta_{0}+\beta_{1}X_{i}+\beta_{2}\left(X_{i}-2010\right)D_{i}+\epsilon_{i},\\ D_{i}=1\left\{X_{i}\geq 2010\right\}. \end{array}$$

In (1), *Y*_*i*_ is an indicator variable for overweight and obesity combined (BMI ≥ 25), obesity (BMI ≥ 30), grades 2 and 3 obesity combined (BMI ≥ 35), or grade 3 obesity (BMI ≥ 40); *X*_*i*_ a continuous variable taking the values of 2000, 2002, 2004, 2006, 2008, 2010, and 2012 for the NHANES waves 1999-2000, 2001-2002, 2003-2004, 2005-2006, 2007-2008, 2009-2010, and 2011-2012, respectively; *D*_*i*_ an indicator variable for *X*_*i*_ ≥ 2010; and *ϵ*_*i*_ the error term. If the estimated coefficient *β*_2_ is significantly different from zero (equivalent to the estimated odds ratio *e* ^*β*_2_^ to be significantly different from one), it indicates the trends of prevalence measures to be different before and after 2010. Piecewise logistic regressions were performed on the overall sample and on each gender and race/ethnicity group, controlling for age group (i.e., 20–39 years old, 40–59 years old, and 60 years and older) and accounting for survey design. All statistical analyses were performed in Stata 13.0 [10].

## 3. Results

In total, 5,560 adults aged 20 years and older participated in the NHANES 2011-2012 wave. Among them, 57 pregnant women and 322 individuals with missing values in body weight/height measures were excluded from the analyses.  Table 1 reports unweighted sample sizes and weighted percentages of the total population. The effective sample size is 5,181, roughly equally split between genders. NHANES oversamples minorities: 1,364 non-Hispanic African Americans and 1,037 Hispanic were included besides non-Hispanic White.

Table 2 reports the estimated BMI and prevalence measures among US adults aged 20 years and older in 2011-2012. The age-adjusted mean BMI was 28.5 (95% CI: 28.0–29.0) among men and 28.8 (28.3–29.3) among women. Non-Hispanic African American women had the highest mean BMI of 32.3 (31.6–33.0) among all gender and race/ethnicity subgroups. The age-adjusted prevalence of overweight and obesity combined (BMI ≥ 25) was 71.1% (68.0%–74.2%) among men and 65.5% (61.8%–69.3%) among women, and the age-adjusted prevalence of obesity (BMI ≥ 30) was 33.3% (30.5%–36.2%) among men and 35.8% (32.3%–39.4%) among women. Among men, 11.8% (9.7%–13.9%) and 4.3% (2.2%–6.3%) were classified in grades 2 and 3 obesity combined (BMI ≥ 35) and grade 3 obesity (BMI ≥ 40), respectively, whereas among women, 16.7% (14.5%–8.9%) and 8.0% (6.6%–9.5%) were classified in grades 2 and 3 obesity combined and grade 3 obesity, respectively.

Substantial disparities in prevalence measures were present across genders and races/ethnicities. Women had higher prevalence of obesity, grades 2 and 3 obesity combined, and grade 3 obesity than men (although the differences were not always statistically significant) among all race/ethnicity groups. Non-Hispanic African American women had the highest prevalence of overweight and obesity combined (81.5%), obesity (56.4%), grades 2 and 3 obesity combined (28.7%), and grade 3 obesity (15.8%) among all gender and race/ethnicity groups, which were 29%, 73%, 92%, and 122% higher than among non-Hispanic White women, respectively.

**Figure 1:**
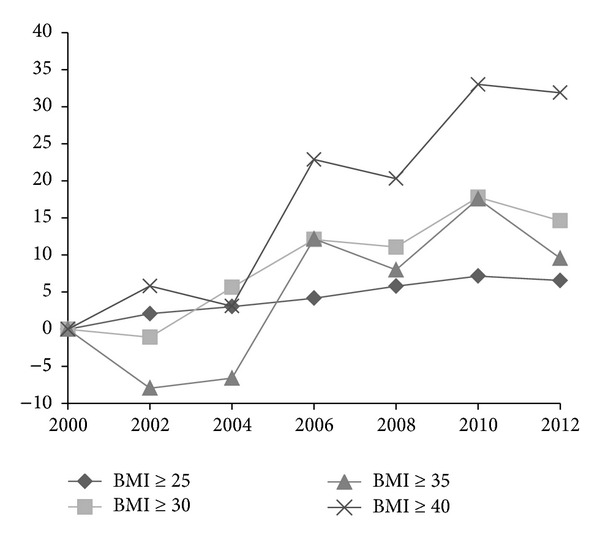
Percentage change in age-adjusted prevalence of overweight and obesity combined (BMI ≥ 25), obesity (BMI ≥ 30), grades 2 and 3 obesity combined (BMI ≥ 35), and grade 3 obesity (BMI ≥ 40) among US adults aged 20 years and older: NHANES 1999-2000 to 2011-2012 (NHANES 1999-2000 as baseline).

Figure 1 shows the percentage change in prevalence measures among US adults of 20 years and older from 1999-2000 to 2011-2012 with 1999-2000 as the baseline. The age-adjusted prevalence of overweight and obesity combined (BMI ≥ 25), obesity (BMI ≥ 30), grades 2 and 3 obesity combined (BMI ≥ 35), and grade 3 obesity (BMI ≥ 40) in 2009-2010 were 7.2%, 17.8%, 17.6%, and 33.0% higher than in 1999-2000. Compared to 2009-2010, the prevalence measures slightly decreased in 2011-2012, but the reductions were not statistically significant at *P* < 0.05.

**Figure 2:**
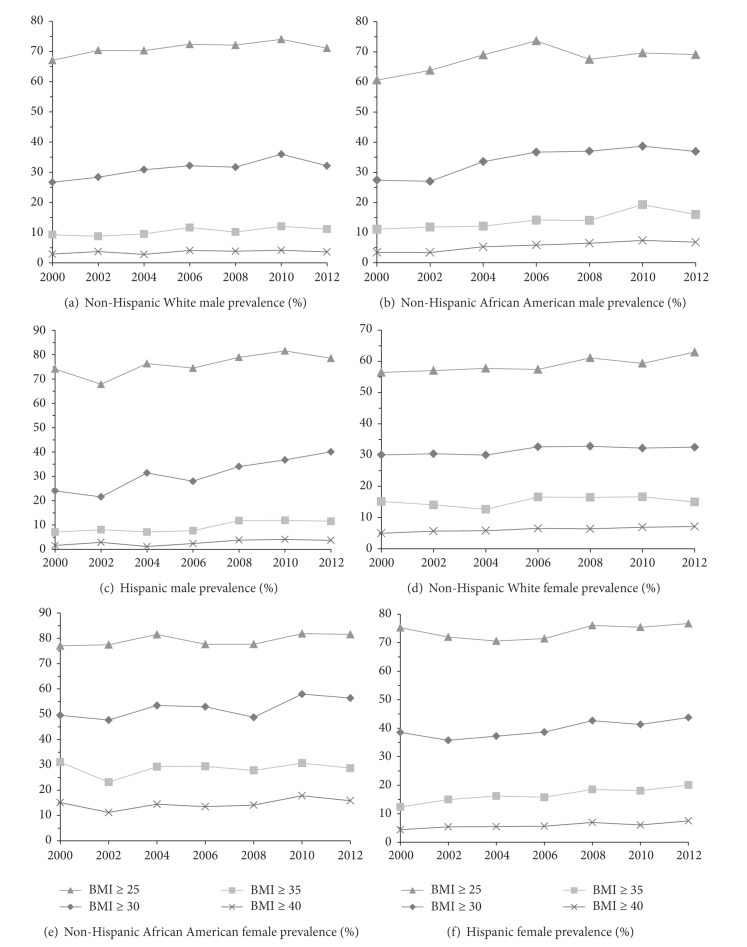
Gender- and race/ethnicity-specific trend in age-adjusted prevalence of overweight and obesity combined (BMI ≥ 25), obesity (BMI ≥ 30), grades 2 and 3 obesity combined (BMI ≥ 35), and grade 3 obesity (BMI ≥ 40) among US adults aged 20 years and older: NHANES 1999-2000 to 2011-2012.

Figure 2 shows the gender- and race/ethnicity-specific trend in the age-adjusted prevalence of overweight and obesity combined (BMI ≥ 25), obesity (BMI ≥ 30), grades 2 and 3 obesity combined (BMI ≥ 35), and grade 3 obesity (BMI ≥ 40) among US adults aged 20 years and older from 1999-2000 to 2011-2012. Compared to 2009-2010, most gender- and race/ethnicity-specific prevalence measures remained unchanged or slightly decreased in 2011-2012, except for the prevalence of overweight and obesity combined among non-Hispanic White women, the prevalence of obesity among Hispanic men, and all 4 prevalence measures among Hispanic women which increased by 1.3 to 3.3 percentage points. None of the changes in gender- and race/ethnicity-specific prevalence measures (24 of them in total) between 2009-2010 and 2011-2012 were statistically significant at *P* < 0.05.

Table 3 reports the statistical tests for trends in obesity over the 14 years of survey cycles from 1999 to 2012 using piecewise logistic regressions. The coefficients are expressed as annualized odds ratios (ORs), denoting the estimated increase per year in the odds of a prevalence measure. A majority of the gender- and race/ethnicity-specific prevalence measures seemed to slightly increase over time (as indicated by the estimated *e* ^*β*_1_^ > 1). For example, the estimated OR for the prevalence of obesity (BMI ≥ 30) among Hispanic women is 1.03 (1.01–1.04), approximately equivalent to a yearly increase in obesity prevalence of 0.6 percentage points. Nearly all gender- and race/ethnicity-specific prevalence measures appeared to deviate downwards from the increasing trend (as indicated by the estimated *e* ^*β*_2_^ < 1) during 2010–2012, but those changes in trends were not statistically significant at *P* < 0.05.

## 4. Discussion

The obesity prevalence in the US increased substantially during the last few decades. The rate of increase appeared to slow down since the 2000s and most recent data on childhood obesity even indicated some leveling off. Using a nationally representative sample, this study estimates the obesity prevalence among US adults aged 20 years and older in 2011-2012 and tracks its trends from 1999 to 2012. The main advantage of NHANES relative to other national health surveillance systems such as the BRFSS and the National Health Interview Survey (NHIS) is the objectively measured body weight and height, which eliminates self-report bias. However, the relatively small sample size of NHANES (about 5,000 in every two-year survey cycle, compared to about 0.4 million a year in BRFSS) limits the precision for population estimates. Unlike BRFSS or NHIS, NHANES is not suited for state-level estimates and between-state comparisons. Moreover, NHANES is a probability sample of the US civilian, noninstitutionalized population, excluding inmates of institutions (e.g., people in penal/mental facilities or homes for the aged, or on active duty in the Armed Forces).

The prevalence measures are based on BMI, a function of weight and height, rather than on body fatness. Although BMI has been found to be closely associated with percentage body fat measured by dual X-ray absorptiometry in the NHANES, these two measures are fundamentally different and their levels of agreement could be a function of gender, age, and race/ethnicity. For instance, percentage body fat was found to be more correlated with BMI in women than men [11]. The relation between percentage body fat and BMI in Hispanic American women differed from that of African American and European American women [12]. A thorough investigation on the differential relationship between BMI and body fatness across population groups and the long-term trend of obesity prevalence measured by percentage body fat is beyond the scope of this study but warranted in future research.

The growth rate of the obesity epidemic among US adults appears to have slowed down in 2000s, but it is still too early to conclude that it has already reached the plateau and begun to level off. Both Figures 1 and 2 showed some decline in the obesity prevalence measures in 2011-2012 compared to in 2009-2010, but none of the changes were statistically significant. This has also been indicated in the results of piecewise logistic regressions where the null hypotheses on the similarity in trends for prevalence measures before and after 2010 were not rejected.

In conclusion, concurrent evidence on the leveling off of the obesity epidemic in the US is thin and the trend is unclear and inconclusive at this time. Given the high prevalence of obesity and its profound socioeconomic consequences, close monitoring of the trend is warranted.

## Figures and Tables

**Table 1:** Unweighted sample sizes and weighted percentages for adults aged 20 years and older: NHANES 2011-2012.

	All races/ethnicities	Non-Hispanic White	Non-Hispanic African American	Hispanic
Both genders	5,181 (100.00%)	1,899 (66.62%)	1,364 (11.48%)	1,037 (14.16%)
Male	2,585 (48.62%)	961 (35.32%)	662 (5.59%)	515 (7.74%)
Female	2,596 (51.38%)	938 (36.89%)	702 (6.85%)	522 (7.62%)

NHANES denotes National Health and Nutrition Examination Survey. Percentage is weighed using NHANES sampling weights to be representative of the US adult population in 2011-2012. The sum of sample sizes for non-Hispanic White, non-Hispanic African American, and Hispanic is not equal to the total sample size because of other races/ethnicities not shown in the table.

**Table 2:** Age-adjusted BMI and prevalence of overweight and obesity combined (BMI ≥ 25), obesity (BMI ≥ 30), grades 2 and 3 obesity combined (BMI ≥ 35), and grade 3 obesity (BMI ≥ 40) among U.S. adults aged 20 years and older: NHANES 2011-2012.

	All races/ethnicities	Non-Hispanic White	Non-Hispanic African American	Hispanic
BMI				
Both genders	28.67 (28.23–29.11)	28.36 (27.78–28.93)	30.83 (30.25–31.40)	29.58 (29.12–30.03)
Male	28.50 (28.04–28.95)	28.36 (27.85–28.88)	28.99 (28.45–29.54)	29.18 (28.68–29.67)
Female	28.84 (28.33–29.34)	28.35 (27.62–29.08)	32.33 (31.63–33.03)	29.94 (29.36–30.52)

BMI ≥ 25 (%)				
Both genders	68.23 (65.07–71.39)	66.95 (63.07–70.83)	75.88 (72.36–79.40)	77.67 (74.20–81.13)
Male	71.11 (68.01–74.21)	71.11 (67.19–75.03)	69.08 (64.79–73.37)	78.51 (73.39–83.63)
Female	65.52 (61.78–69.27)	62.97 (57.97–67.98)	81.54 (77.81–85.27)	76.70 (73.16–80.23)

BMI ≥ 30 (%)				
Both genders	34.63 (31.79–37.47)	32.36 (28.71–36.00)	47.63 (44.25–51.00)	42.05 (38.63–45.48)
Male	33.34 (30.51–36.17)	32.15 (29.32–34.97)	36.95 (33.13–40.77)	40.07 (35.65–44.49)
Female	35.81 (32.26–39.36)	32.55 (26.81–38.28)	56.38 (52.14–60.60)	43.73 (39.48–47.97)

BMI ≥ 35 (%)				
Both genders	14.31 (12.52–16.11)	13.12 (10.64–15.60)	23.02 (20.70–25.35)	15.89 (13.47–18.30)
Male	11.80 (9.65–13.94)	11.19 (8.74–13.64)	16.03 (12.53–19.52)	11.56 (9.12–14.01)
Female	16.69 (14.48–18.91)	14.98 (11.76–18.21)	28.73 (25.66–31.80)	20.05 (16.80–23.30)

BMI ≥ 40 (%)				
Both genders	6.19 (4.85–7.53)	5.41 (3.62–7.19)	11.76 (9.99–13.53)	5.67 (4.49–6.85)
Male	4.27 (2.23–6.32)	3.64 (1.12–6.16)	6.86 (5.17–8.54)	3.73 (2.54–4.91)
Female	8.04 (6.61–9.46)	7.13 (5.11–9.15)	15.84 (12.87–18.87)	7.49 (5.75–9.23)

BMI denotes body mass index defined by body weight in kilograms divided by height in meters squared. NHANES denotes National Health and Nutrition Examination Survey. Prevalence is weighed using NHANES sampling weights to be representative of the U.S. adult population in 2011-2012. Age is adjusted by direct standardization to the year 2000 Census population using the age groups 20–30 years old, 40–59 years old, and 60 years and older. 95% confidence interval is in parenthesis.

**Table 3:** Estimated annual change in the odds of the prevalence of overweight and obesity combined (BMI ≥ 25), obesity (BMI ≥ 30), grades 2 and 3 obesity combined (BMI ≥ 35), and grade 3 obesity (BMI ≥ 40) among U.S. adults aged 20 years and older using piecewise logistic regressions: NHANES 1999-2000 to 2011-2012.

	All races/ethnicities		Non-Hispanic White		Non-Hispanic African American		Hispanic	
	*e* ^*β*_1_^	*e* ^*β*_2_^	*e* ^*β*_1_^	*e* ^*β*_2_^	*e* ^*β*_1_^	*e* ^*β*_2_^	*e* ^*β*_1_^	*e* ^*β*_2_^
BMI ≥ 25								
Both genders	1.020** (1.006–1.034)	0.972 (0.892–1.060)	1.021* (1.002–1.040)	0.981 (0.884–1.089)	1.032*** (1.015–1.049)	0.953 (0.849–1.069)	1.035** (1.011–1.059)	0.963 (0.845–1.098)
Male	1.030** (1.012–1.049)	0.902 (0.814–1.000)	1.029* (1.005–1.053)	0.905 (0.796–1.029)	1.041*** (1.021–1.063)	0.899 (0.794–1.018)	1.055** (1.022–1.089)	0.894 (0.732–1.092)
Female	1.012 (0.995–1.029)	1.033 (0.939–1.136)	1.016 (0.992–1.040)	1.042 (0.920–1.179)	1.020 (0.992–1.049)	1.021 (0.867–1.201)	1.013 (0.984–1.043)	1.045 (0.908–1.203)

BMI ≥ 30								
Both genders	1.027*** (1.013–1.040)	0.957 (0.889–1.030)	1.026** (1.009–1.043)	0.942 (0.855–1.039)	1.039** (1.017–1.061)	0.953 (0.857–1.060)	1.038** (1.014–1.061)	0.908 (0.822–1.002)
Male	1.040*** (1.022–1.058)	0.926 (0.847–1.013)	1.057*** (1.030–1.084)	0.892 (0.786–1.011)	1.060*** (1.028–1.093)	1.050 (0.903–1.221)	1.015 (0.995–1.034)	0.977 (0.847–1.127)
Female	1.015* (1.000–1.029)	0.986 (0.903–1.076)	1.027 (0.999–1.056)	1.001 (0.876–1.143)	1.019 (0.993–1.046)	1.007 (0.890–1.139)	1.026** (1.010–1.041)	0.942 (0.864–1.026)

BMI ≥ 35								
Both genders	1.034** (1.009–1.060)	0.953 (0.839–1.083)	1.028* (1.006–1.051)	0.941 (0.853–1.039)	1.054*** (1.031–1.078)	0.959 (0.857–1.072)	1.033* (1.002–1.064)	0.930 (0.796–1.085)
Male	1.020* (1.004–1.037)	0.933 (0.851–1.024)	1.062** (1.022–1.103)	0.907 (0.760–1.084)	1.063** (1.023–1.105)	0.948 (0.787–1.141)	1.023* (1.001–1.046)	0.921 (0.797–1.063)
Female	1.027** (1.007–1.048)	0.923 (0.817–1.044)	1.009 (0.983–1.036)	0.970 (0.859–1.095)	1.054*** (1.029–1.080)	0.952 (0.840–1.080)	1.031** (1.010–1.053)	0.972 (0.858–1.101)

BMI ≥ 40								
Both genders	1.031** (1.010–1.053)	0.972 (0.858–1.101)	1.034* (1.005–1.063)	0.950 (0.787–1.146)	1.045* (1.016–1.075)	0.928 (0.817–1.054)	1.066** (1.0165–1.118)	0.953 (0.801–1.132)
Male	1.038* (1.001–1.076)	0.958 (0.736–1.247)	1.034 (0.990–1.079)	0.912 (0.625–1.329)	1.088** (1.038–1.141)	0.876 (0.710–1.080)	1.092* (1.014–1.175)	0.892 (0.678–1.175)
Female	1.028* (1.005–1.051)	0.980 (0.873–1.100)	1.035* (1.003–1.068)	0.975 (0.818–1.162)	1.030 (0.996–1.065)	0.963 (0.822–1.129)	1.053* (1.003–1.105)	0.981 (0.811–1.186)

See (1) for model setup. Odds ratio and 95% confidence interval are reported. *P < 0.05, **P < 0.01, and ***P < 0.001.
